# A model for germ cell development in a fully segmented worm

**DOI:** 10.1186/s40851-015-0035-y

**Published:** 2015-12-07

**Authors:** Mercedes Maceren-Pates, Yoshihisa Kurita, Gaudioso Pates, Michiyasu Yoshikuni

**Affiliations:** Fishery Research Laboratory, Kyushu University, 4-46-24 Tsuyazaki, Fukutsu, 811-3304 Japan; Graduate School of Agricultural Science, Tohoku University, 15 Mukai, Konorihama, Oshika, Miyagi 986-2242 Japan

**Keywords:** *Vasa*, *Perinereis nuntia*, Parapodium, Primordial germ cell, Gonad, Septum

## Abstract

**Introduction:**

Polychaetes are segmented marine worms with body segments separated by a complete or incomplete septum. In most polychaetes the whole body cavity is filled with gametes during the breeding season. *Platynereis dumerilii (Pl. dumerilii)*, which has an incomplete septum was shown to develop a single gonadal structure for gamete production located in the neck region. However, in *Perinereis nuntia* (*Pe. nuntia*), which has a complete septum separating each segment, the developmental feature of gametes remains unknown. To clarify this, the marker gene *vasa* was used to trace the development of germ cells throughout the life stages of *Pe. nuntia*.

**Results:**

In three-segmented juveniles, *Pn-vasa* was expressed in the parapodia and in the two cells localized in the pygidium. During the addition of a new segment, *Pn-vasa* positive cells in the pygidium increased from two to four and two new *Pn-vasa* positive cells were found in the newly-generated segment. In adults, *Pn-vasa* was expressed in a large cell cluster at the distal end of the parapodia, in smaller cell clusters (which had an elongated form in the trunk area of the parapodia), and in oocytes in the coelomic cavity. This may suggest that germ cells settle in the parapodia and later translocate into the coelomic cavity to develop into oocytes.

**Conclusion:**

Our observations will help in understanding the mechanism of germ cell development in all body segments of *Pe. nuntia*. We hypothesize that primordial germ cells are supplied from the pygidium to every newly-generating segment which later settle in the parapodium. This will explain how polychaetes can generate gametes in each body segment, even those that are independently separated with a complete septum.

**Electronic supplementary material:**

The online version of this article (doi:10.1186/s40851-015-0035-y) contains supplementary material, which is available to authorized users.

## Introduction

Annelids, the segmented worms, are one of the largest and most widely distributed animal phyla. Recent phylogenetic reconstruction of this phylum shows Chaetopteridae, Myzostomida, and Sipuncula branching off from the basal nodes, while the remaining taxa are classified into two clades: Errantia and Sedentaria [1, 2]. On the basis of this reconstruction, the ancestral annelid is characterized by a serial division of the body into numerous similar structures also known as homonomous segmentation [1, 3, 4]. Besides segmentation, the presence of a pygidium, pygidial cirri, biramous parapodia and large prostomium are characteristics of the basal taxa [3].

The Nereidid polychaetes (belonging to Errantia) are benthic marine worms that show an interesting strategy of reproduction through a process called epitoky. This process involves the transformation of an immature worm (atoke) into a sexually mature form (epitoke) with specialized swimming and sensory ability which increase the chances of reproductive success [5–7]. All body segments of the epitoke are completely filled with gametes which are released during reproductive swimming. This unique reproductive strategy has caught the attention of many biologists working on Nereidids, making it a model animal for reproductive studies.

The reproductive biology of the Nereidids has long been studied not only for the biological aspects but also for the conservation and propagation of the species [6–11]. However, most studies are limited to only a few Nereidid species and mostly focus on *Platynereis dumerilii* (*Pl. dumerilii*). *Platynereis dumerilii* has currently emerged as the leading model animal in the clade Errantia and is described as a homonomous segmented species [1, 3] but with incomplete inter-segmental septa [12]. A recent study using a molecular germline marker to trace gamete generation in this species showed that germ cells originate from the mesodermal posterior growth zone (MPGZ) and migrate into the anterior segments to form a transverse cluster of cells in the neck region. This region was then referred to as the primary gonad which produces gamete to fill a large part of the body [13].

Unlike the well-studied *Pl. dumerilii*, the gamete production mechanism in other Nereidid groups, that have body segments separated by complete inter-segmental septa, has never been identified. This group of polychaetes may have developed some unique mechanisms for gamete production in each body segment, which are different from that of *Pl. dumerilii*. Further study of this mechanism, together with the mechanism already observed in other groups with incomplete septa such as *Pl. dumerilii*, will provide a better understanding of the ancestral mechanism of gamete production in Annelids.

Our results suggest a new mechanism for gamete production in *Pe. nuntia*, using *vasa* as a putative germ cell marker. The *Pn-vasa* signal was detected in all body segments during the growth of *Pe. nuntia* from larva to adult. The site of expression changed from the distal end of the parapodium to the inner coelomic cavity in accordance to the growth of segments. We hypothesize that primordial germ cells (PGCs) are supplied from the pygidium to every newly-generating segment.

## Materials and methods

### Animals

The Nereidid polychaete worms, *Pe. nuntia*, were purchased from the commercial hatchery in Oita Prefecture, Japan. They were maintained in the Kyushu University Fishery Research Laboratory based on the culture methods employed by the hatchery. Embryos that were raised in plastic containers supplied with running filtered seawater were observed during their developmental stages as described in [8] and [14]. The identification of this species was based on the taxonomic descriptions provided by Glasby and Hsieh [15].

### cDNA cloning and molecular phylogeny of *Pn-vasa* gene

The total RNA was extracted from the unfertilized eggs of *Pe. nuntia* using the RNeasy Plant Mini kit (Qiagen). The complementary DNA was generated from this RNA using a PrimeScript RT-PCR kit (Takara). Two degenerate primers (forward: 5′-atcaactttgacaaatacga-3′; reverse: 5′-gcgctgaacatsagygtctg-3′) were designed based on the *Pdu-vasa* mRNA sequence reported by Rebscher et al. [13] (Genbank Acc. No. AM048812.1). Primers were designed corresponding to the highly conserved regions within the coding region of the *vasa* transcript. The resulting PCR product (619 bp) was cloned into pGEMT-easy vector (Promega) and sequenced. Homology searches were performed using BLASTn in the NCBI database.

Molecular phylogenetic analysis was performed as follows: Related sequences were retrieved from public databases based on BLAST searches and prior knowledge. Multiple alignments of related amino acid sequences were created and a phylogenetic tree was constructed by maximum likelihood using the WAG + I model selected by the Akaike Information Criterion. Alignment, model selection and tree construction was performed with MEGA 5.0 [16]. Five hundred bootstrap pseudo-replicates were performed to evaluate the confidence for each node. PL10 genes from various groups of species were included in the analysis as an out-group.

### *In situ *hybridization

*Pn-vasa* plasmid clones were used as a template to synthesize both sense and anti-sense RNA probes by *in vitro* transcription using the DIG RNA labeling kit (Roche). Juveniles and adult worms were relaxed for 2–5 min in 0.3 % ethylene glycol monohexyl ether in seawater and then fixed overnight at 4 °C in 4 % formaldehyde in phosphate buffered saline (PBS). The fixative was rinsed off by washing twice with PBS, and specimens were dehydrated in a series of methanol in PBS and stored in −20 °C until use. Whole-mount in situ hybridization was performed as described in the polychaete *Chaetopterus* [17] with some modifications. Embryos and larvae were treated in the same way as the juvenile and adult worms except that anesthetic was omitted. Proteinase K treatment was reduced from 20 min at 37 °C in juveniles and adults to 5 min at room temperature in embryos and 10 min at room temperature in larvae. After *in situ *hybridization, an immuno-reaction was induced using the anti-DIG alkaline phosphatase conjugated Fab fragment (Roche) and visualized by the corresponding substrate, Nitro Blue Tetrazolium/5-Bromo-4-chloro-3-indolyl phosphate (NBT/BCIP).

### Histology

Hybridized adult worms were fixed with 4 % paraformaldehyde overnight at 4 °C, dehydrated in series of ethanol, embedded in the paraffin and sliced into 7 μm thick sections. The sections were then deparaffinized, mounted, analyzed and photographed under the microscope. Another set of adult worm samples were also prepared for normal hematoxylin and eosin (H and E) stain following the same procedure as mentioned above, except that samples were fixed with Davidson’s fixative instead of 4 % paraformaldehyde.

## Results

To determine the spatial and temporal expression patterns of *Pn-vasa* in the Nereidid *Pe. nuntia* at different developmental stages, we performed a whole-mount *in situ *hybridization experiment using *vasa* as a molecular germline marker.

*Vasa* is an ATP-dependent RNA helicase gene of the DEAD-box family, essential for germ cell development [18–23]. This gene was originally identified in *Drosophila* and was revealed to have a highly conserved role among different organisms [24–39]. Since then, *vasa* has been used as a molecular marker to study germ cell development in many animals including polychaetes [13, 32–34]. To date *vasa* expressed not only in germ cells but also in somatic stem cells as well [40–42].

### *Pn-vasa* cDNA cloning and phylogenetic analysis

**Fig. 1:**
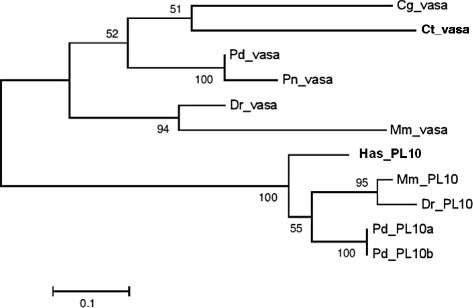
Phylogenetic analysis of *Pn-vasa*. Phylogenetic tree constructed by a maximum likelihood method showing orthology of *Pn-vasa*. Species abbreviations: Cg, *Crassostrea gigas*; Ct, *Capitella teleta*; Pd, *Platynereis dumerilii*; Pn*, Perinereis nuntia*; Dr, *Danio rerio*; Mm, *Mus musculus*; Has, *Haliotis asinina*. The number on the nodes corresponds to the bootstrap support values. Distance scale represents the number of differences between sequences (e.g. 0.1 means 10 % differences between two sequences)

A 619 bp cDNA fragment was amplified, cloned and sequenced from the unfertilized egg of the mature worm and used as an anti-sense probe for the *in situ *hybridization experiment. The amino acid sequence showed 90 % identity to a previously reported complete sequence of *Pl. dumerilii vasa* mRNA [13] and more than 60 % sequence identity to other known *vasa* homologues from different annelid species such as in *Urechis unicinctus* (JQ665715.1), *Capitella teleta* (BK006523.1), *Enchytraeus japonensis* (AB306293.1) and *Tubifex tubifex* (AB257139.1). Molecular phylogenetic analysis showed that *Pn-vasa* formed a monophylogenetic clade with *vasa* homologues of other species and was closely related to the DEAD box helicase *PL10* genes (Fig. 1).

### *Pn-vasa* expression patterns in adult during oogenesis

**Fig. 2:**
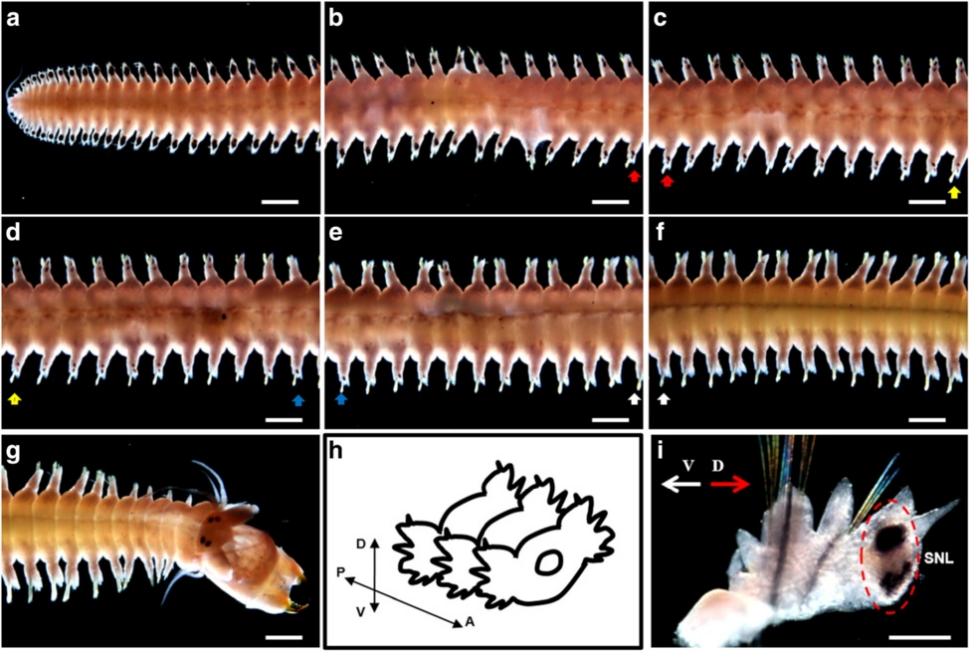
*Pn-vasa* expression patterns in the adult *Pe. nuntia*. **a**–**g** *In situ* hybridization experiment showing the *Pn-vasa* expressions in the parapodia of each body segment, dorsal view. **h** A schematic representation showing the direction of the parapodium. **i** A single parapodium from the posterior-body region showing the location of the *Pn-vasa* signal in the SNL (broken circle). Arrows (**b**–**f**) with the same colors below the parapodium represent the same segment. D, dorsal direction; V, ventral direction; P, posterior direction; A, anterior direction; SNL, superior notopodial ligule. Scale bar: **a**–**g** (1 mm); I (200 μm)

In the adult Nereidid polychaete worm, *Pn-vasa* mRNA was detected in the dorsal parapodia on both sides of nearly all body segments. The intensity of the *Pn-vasa* signal was strongest at the most posterior-region and gradually decreased along the mid-body until no signal was detected towards the most anterior-region (Fig. 2a–g). Interestingly, *Pn-vasa* was only detected in the superior notopodial ligule (SNL) of the parapodium (Fig. 2i).

**Fig. 3:**
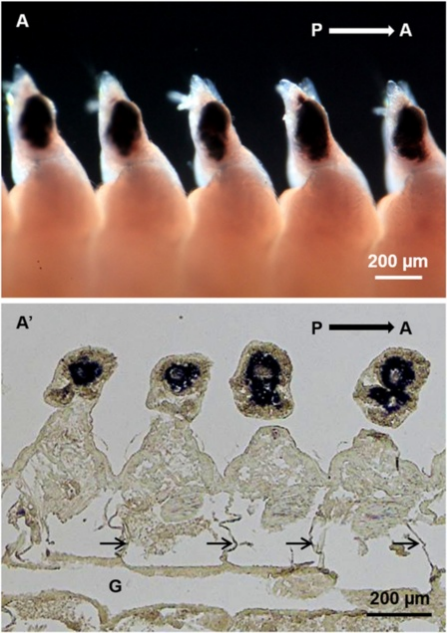
*Pn-vasa* mRNA in the parapodia of the posterior-body region of the adult worm. **A** Hybridized worm showing the *Pn-vasa* strong expressions in the parapodia, dorsal view. **A’** Coronal section of (**A**) showing the position of a big cell cluster in each parapodium. Arrows indicate the septum. P, posterior direction; A, anterior direction; G, gut

**Fig. 4:**
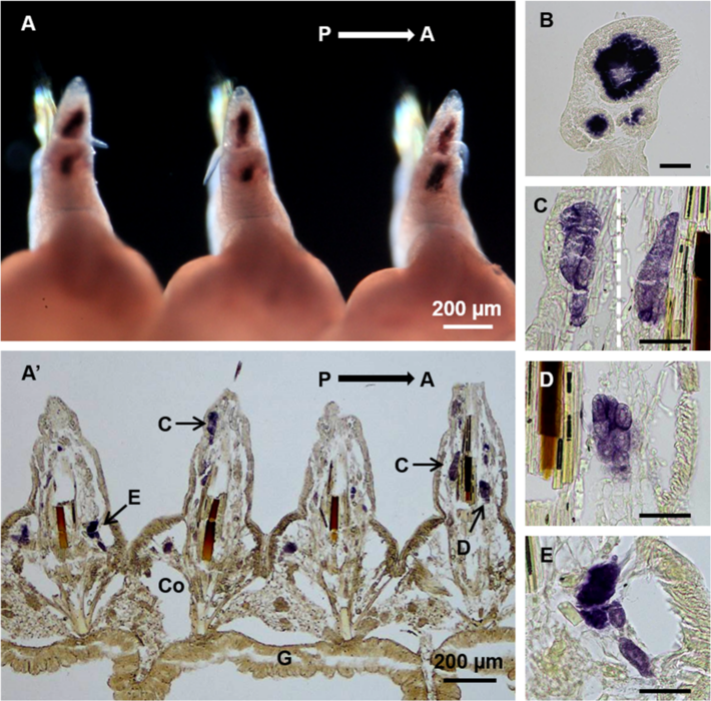
*Pn-vasa* mRNA in the parapodia of the mid-body region of the adult worm. **A** Hybridized worm showing the weak *vasa* expressions in the parapodia, dorsal view. **A’** Coronal section of (**A**) showing the distribution of the *Pn-vasa* positive cells in the parapodia and in the coelomic cavity (Co). **B** A single parapodium showing a large cell cluster and smaller cell clusters. **C**–**E** Cell clusters of various forms with *Pn-vasa* signals. P, posterior direction; A, anterior direction; G, gut. Scale bar: **B**–**E** (50 μm)

**Fig. 5:**
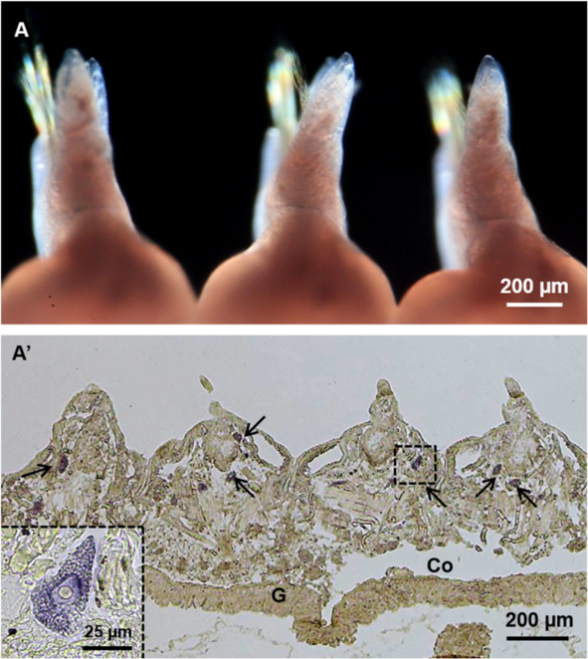
*Pn-vasa* mRNA in the parapodia of the anterior-body region of the adult worm. **A** Hybridized worm with no apparent *Pn-vasa* expression in the parapodia, dorsal view. **A’** A coronal section of (**A**) showing the distribution of *Pn-vasa* positive cells (arrows, inset) in the coelomic cavity (Co). G, gut

Histological examinations revealed that in the posterior-body region, *Pn-vasa* was strongly expressed in a population of cells in each parapodium (Fig. 3A, A’). In the mid-body region, the *Pn-vasa* signal was detected in cell clusters of various forms based on their location from the distal end of the parapodium to the coelomic cavity (Fig. 4A, A’). Most of these cell clusters had an elongated form with strong *Pn-vasa* signals throughout the entire cell (Fig. 4C–E). In the anterior-body region, *Pn-vasa* was detected in single cells (Fig. 5A’, arrows) that had strong *Pn-vasa* signals throughout the entire cell (Fig. 5A’, inset). Most of these cells were found in the coelomic cavity (Fig. 5A’).

**Fig. 6:**
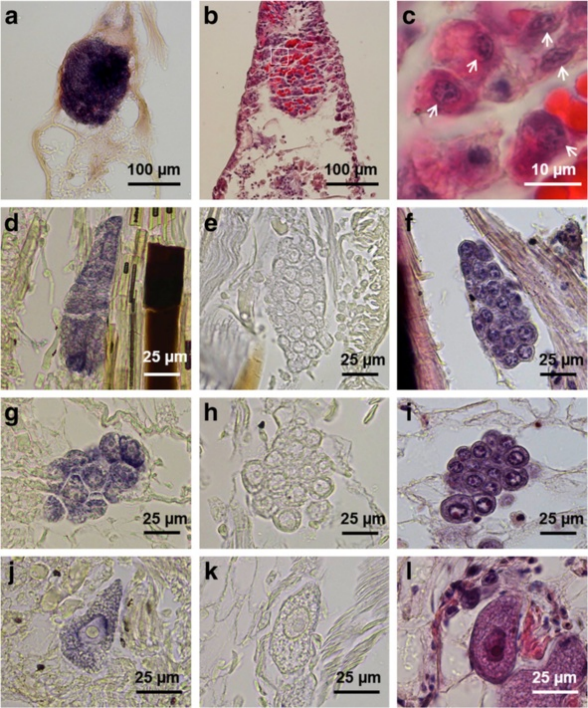
Germ cell development of *Pe. nuntia*. **a** Hybridized slide section showing the *Pn-vasa* expression in the big cell cluster at the distal end of the parapodium. **d**,**g**, **j** *Pn-vasa* positive cells observed from the trunk area of the parapodium down to the coelomic cavity. **b** Thin section (5 μm) of the parapodium stained with hematoxylin and eosin showing the big cell cluster. **c** Cells from figure **b** (box frame) at higher magnification (1,000×) showing the cell’s condensed chromosomes (arrows) and pink-colored granular-like structures. **f**, **i**, **l** The corresponding hematoxylin and eosin stain of the *Pn-vasa* positive cells. Condensed chromosomes are visible in cell clusters (**f** and **i**). **e**, **h**, **k** Sense control experiment. Note: Figure **d** is taken from Fig. 4C, while figure **g** is taken from other parapodium source. Figure **k** is also taken from Fig. 5 (inset)

To determine the stages of *Pn-vasa* positive cells (Fig. 6a, d, g, j), we conducted a histological observation of the cells using hematoxylin and eosin stains (Fig. 6c, f, i, l). In the distal area of the parapodium, the big cell cluster (Fig. 6a–b) is composed of cells with condensed chromosomes and pink-colored granular-like structures (Fig. 6c). In the trunk area of the parapodium, the cell clusters had an elongated shape, while the cells inside the cluster also contained condensed chromosomes (Fig. 6f, i). In the coelomic cavity, the cells were larger than those cells in the parapodial area with no visible chromosomes in the nucleus (Fig. 6l). The corresponding sense control experiment is shown in Fig. 6e, h, k .

### *Pn-vasa* expression patterns in embryos and larvae

**Fig. 7:**
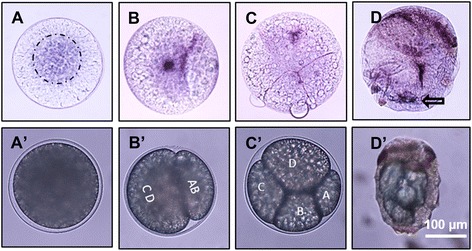
*Pn-vasa* mRNA in embryos and larva. **A** Fertilized egg showing the *Pn-vasa* signal in the surface area, top view. **B** At 2-cell stage, *Pn-vasa* is expressed in the CD-cell quadrant. **C** At 4-cell stage, *Pn-vas*a is only expressed in the D-cell quadrant. **D** At 65 hpf, dorsal view, *Pn-vasa* is strongly expressed in the most posterior region (black arrow). The anterior region of the larva is characterized by the presence of a mesodermal band which contains the pigment cells [46]. Scale bar: **A**–**D** (100 μm)

**Fig. 8:**
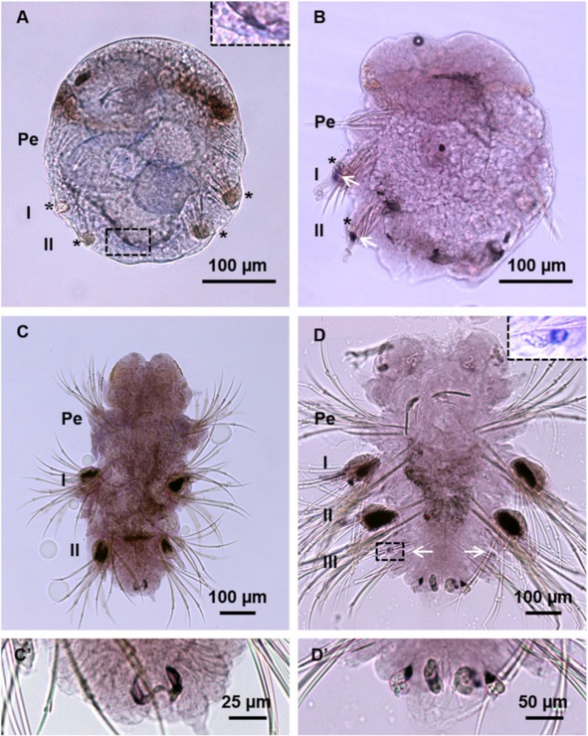
*Pn-vasa* expression patterns in larva and juvenile. **A** Larva at 72 hpf showing the presence of four somatic cell clusters in segments I and II (asterisks) and a small *Pn-vasa* positive cell at the most posterior end (box frame, inset). **B** In the 3-segmented worm at 4 dpf, *Pn-vasa* positive cells (arrows) found closer to the somatic cell clusters (asterisks) at the anlage of the parapodia. **C** At 5 dpf, *Pn-vasa* is expressed in the parapodia in segments I and II and *Pn-vasa* also expressed in two cells in the pygidium (C’). **D** In the 4-segmented worm, four *Pn-vasa* positive cells are found in the pygidium (D’) and two small *Pn-vasa* positive cells are also found on both sides of the newly generated segment (**D** arrows; inset). No signal found in the future peristomium (Pe). Note: the right side of the parapodial segments in figure **B** is behind the worm’s body. In figure **D** (inset), the cell is taken using higher magnification (400×) and at different focal plane

During embryogenesis, *Pn-vasa* mRNA was detected in the surface area of the fertilized egg (Fig. 7A, A’). It was then detected in the CD cell at the two-cell stage (Fig. 7B, B’) and in the D cell at the four-cell stage (Fig. 7C, C’). At 65 h post fertilization (hpf), *Pn-vasa* signal was observed in the posterior end of the larva (Fig. 7D; black arrow). At 72 hpf, we observed four somatic cell clusters in a spherical shape (Fig. 8A, asterisks) at the base of each parapodium in segments I and II (Fig. 8A). At the same time, we also observed the emergence of a *Pn-vasa*-positive cell at the posterior end of the larva (Fig. 8A, box frame). At 96 hpf, *Pn-vasa* positive cells were found closer to the somatic cell clusters (Fig. 8B, asterisks) in segments I and II.

### *Pn-vasa* expression patterns in nectochaete larvae and juveniles

**Fig. 9:**
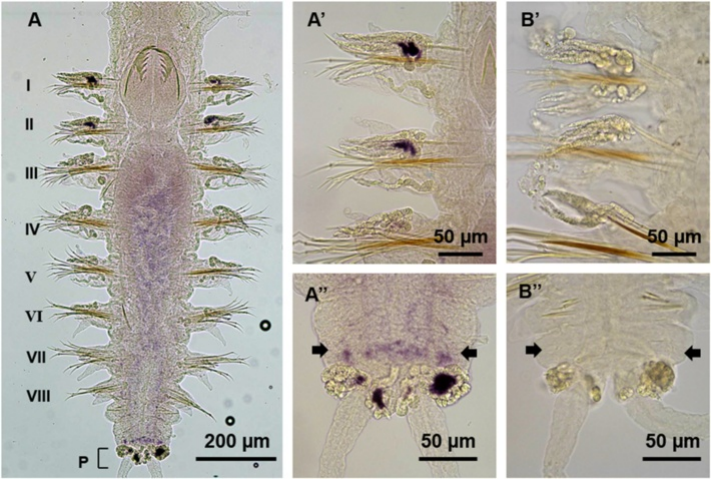
*Pn-vasa* mRNA in the juvenile worm. **A** *Pn-vasa* signal is strongly expressed in the older segments, I and II and becomes weaker towards the younger segments (Segment III-VIII). **A’** Parapodia of (**A**) showing the strong hybridization signals in older segments I and II with the sense probe experiment B’. **A** **”** Posterior end of (**A**) showing the more intense *Pn-vasa* signals in the pygidium (P) and another *Pn-vasa* signals detected in the anterior border of the pygidium (A”, arrows). (B”) Sense probe experiment. Pe, peristomium or Ce, cephalic segment

In 3-segmented nectochaete worms (6 days post fertilization, dpf), *Pn-vasa* was detected in the parapodia on both sides of segments I and II, at the exact place where the *Pn-vasa*-positive cells were found closer to the somatic cell cluster during the larval stage (Fig. 8C, D). No *Pn-vasa* signal was detected in the future peristomium segment (Fig. 8C, D). At this stage, we also observed the emergence of two *Pn-vasa*-positive cells positioned in the pygidium (Fig. 8C, C’). In 4-segmented nectochaete worms (8 dpf), four *Pn-vasa* positive cells were observed in the pygidium (Fig. 8D, D’) while two other *Pn-vasa* positive cells were found on both sides of the newly-generated segment (Fig. 8D, box frame; inset). In 8-segmented juveniles (10 dpf), *Pn-vasa* signal in the parapodia was stronger in the older segments (segments I and II) and became weaker towards the younger segments (segments III-VIII) (Fig. 9A). Another *Pn-vasa* signal was also observed in the anterior border of the pygidium aside from the *Pn-vasa* positive cells localized in the pygidium (Fig. 9A”, arrows).

## Discussion

### *Pn-vasa* expression in the parapodia of *Pe. nuntia*

*Pn-vasa* mRNA was expressed in the parapodia, where signal intensity changed according to the growth of the segments. More intense signals were found in the younger parapodia of the posterior-body region and gradually decreased along the mid-body until a signal was undetectable in the anterior-body region. The strong *Pn-vasa* signal in the parapodia of the posterior region was due to the large aggregates of cell clusters (Fig. 3A’) in each parapodium. From the posterior to mid-body regions, these large cell clusters in the parapodia appeared to divide into smaller cell clusters (Fig. 4A, arrows C–E in Fig. 4A’). The more elongated form and the position of these smaller clusters are consistent with the idea that the clusters migrate from the distal areas of the parapodia to the coelomic cavity where they began the process of oogenesis (Fig. 4B–E). In the anterior-body region (Fig. 5A, A’), nearly all cell clusters in the parapodia had already disappeared, however, *Pn-vasa* signals were observed in single cells that looked like oocytes in the coelomic cavity (Fig. 5A’, arrows; inset). These series of observations may suggest that PGCs settle in the parapodium as a single cell, proliferate to form a large germ cell cluster (Fig. 3A, A’), separate from a large cell cluster into smaller clusters (Fig. 4A, A’) and migrate into the coelomic cavity to develop into oocytes (Fig.5A’).

The existence of a germ cell production site in the Nereidid polychaete was reported in *Pl. dumerilii* [13] where one primary gonad was found located in the neck region. In contrast, in the present study, the large cell cluster with a strong *Pn-vasa* signal was observed in the parapodia of each body segment. The presence of these cell clusters in the parapodia may indicate the presence of a gonad of *Pe. nuntia*. Although the molecular mechanism underlying the difference in gonad location between these two closely-related species is not yet known, this may be morphologically related to the difference in their septum formation. In *Pl. dumerilii*, the incomplete septum [12] allows the translocation of PGCs to the neck region, and, subsequently, expansion of germ cells in the whole coelomic cavity as one common compartment [13]. While on the other hand, *Pe. nuntia* has a complete septum between segments (Additional file 1: Figure S1), thus, each segment has to be equipped with sites for germ cell generation. The original settling area of *Pn-vasa* positive cells in each parapodium may act as the primary gonad of *Pe. nuntia*.

### Expression of *Pn-vasa* in the embryo of *Pe. nuntia*

It has been shown that the modes of maternally supplied *vasa* mRNA distribution in the early stages of embryo vary among animals [23, 30, 32–35]. Here, we observed that this mode also varies in a closely related species in polychaetes. In *Pl. dumerilii vasa* mRNA was uniformly distributed in early cleavage stages and later found in the micromeres, including the mesoblast 4D at 38-cell stage [13]. In the present study, *Pn-vasa* signal appeared to be restricted in the surface area of the fertilized egg (Fig. 7A). This may be the area described as yolk-free cytoplasm in *Pl. dumerilii* [13]. During the four cell stage, *Pn-vasa* mRNA was localized into the D cell quadrant, a similar pattern of expression observed in other polychaete species such as *Tubifex tubifex* [32]. At 65 hpf, *Pn-vasa* signal was then localized at the posterior end of the larva (Fig. 7D, arrow). This posterior end was described as the MPGZ in *Pl. dumerilii*, where *Pdu-vasa* was also localized at 48 hpf [13].

### Germ cell development in *Pe. nuntia*

In *Pl. dumerilii*, PGCs migrate from the MPGZ towards the primary gonad in the neck region during the late larval stage [13]. In the present study, we did not observe any *vasa* signal in the neck region nor traversing cells with *vasa* signals from the posterior region (Figs. 8 and 9). Instead, we observed *Pn-vasa* positive cells located beside the somatic cell clusters at the anlage of the parapodia during the early larval stage (Fig. 8B, arrows). In 3-segmented nectochaete worms, two *Pn-vasa* positive cells were observed in the pygidium (Fig. 8C, C’). During the addition of a new segment, four *Pn-vasa* positive cells were observed in the pygidium (Fig. 8D, D’) while two other *Pn-vasa* positive cells were found in the newly-generated segment (Fig. 8D, arrows; inset). These observations may indicate that the *Pn-vasa* positive cells divide in the pygidium and daughter cells are supplied to both parapodia of the newly-generated segment.

In many animals including polychaetes, *vasa* has been reported to express in the somatic stem cells [22, 27, 40–42] which has led to a hypothesis that both PGCs and stem cells share some common molecular signatures [40–44]. In *Pl. dumerilii*, *Pdu-vasa* was shown to express in these two distinct cell populations: PGCs and somatic stem cells, in the MPGZ during the larval stage [13, 42]. This MPGZ was also described as the segment addition zone [40] or segment/pygidium boundary [45] in other reports. Only PGCs from this zone migrate to the neck region and form the germline of *Pl. dumerilii* [13]. Whereas somatic stem cell populations are believed to be responsible for the formation of new segments [40]. In the present study, we also observed *Pn-vasa* signals in the boundary area between the newly-generated segment and the pygidium during the juvenile stage in addition to *Pn-vasa* positive cells localized in the pygidium (Fig. 9). With this observation, we hypothesize two possible mechanisms for the appearance of *Pn-vasa* positive cells in the parapodium. One possibility may be that *Pn-vasa* positive cells from the pygidium migrate to the MPGZ and settle in the parapodium of the newly-generated segment. The MPGZ may act as a queuing area for *Pn-vasa* cells and may double as an area for somatic stem cell populations to generate new segments. A second possibility is the spontaneous emergence of *Pn-vasa* positive cells in every new segment without translocation of germ cells from the pygidium. To confirm our hypothesis, we need to perform a more detailed histological observation and analysis using other molecular markers to determine the identity of the cells.

## Conclusion

**Fig. 10:**
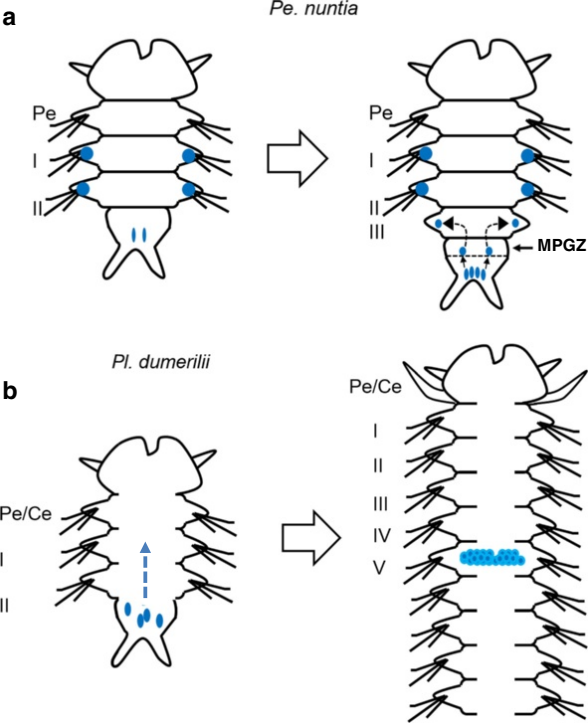
Schematic diagrams on the mechanisms of germ cell distribution in the Nereidid polychaetes. **A** Hypothetical germ cell distribution in *Pe. nuntia* (with complete inter-segmental septa) from the pygidium into each newly-generating segment. **B** Germ cell distribution in *Pl. dumerilii* (with incomplete inter-segmental septa) from the MPGZ towards the neck region (fifth segment) during the juvenile stage II [13]

This study proposed a new mechanism for germ cell development in every segment separated by a complete septum in *Pe. nuntia. Pn-vasa* was found to express in the cells of the pygidium suggesting that these cells serve as the main source of germ cells. These germ cells are then supplied to every newly-generating segment. This is different from the previously reported mechanism of gamete production in *Pl. dumerilii*. The difference in the gamete production pattern of these two homonomous segmented species appears to be related to the presence or absence of a complete septum between segments (Fig. 10). These results provide a fundamental basis for the understanding of the evolutional change in gamete production of polychaetes.

## Additional file

Additional file 1: Figure S1. The morphology of the Inter-segmental septa in the adult *P. nuntia*. Sections show the presence of complete inter-segmental septa (S) in the tail (A), mid-body (B) and anterior-body region (C). Coronal sections, 7 μm; hematoxylin and eosin stain. (PNG 2009 kb)

## Abbreviations

MPGZ: mesodermal posterior growth zone
PGC: primordial germ cell
SNL: superior notopodial ligule
