# Supplementary figures and images for: A model for germ cell development in a fully segmented worm
Source: Zoological Lett. 2015 Dec 7;1:34. doi: 10.1186/s40851-015-0035-y (PMC4672553; doi:10.1186/s40851-015-0035-y)

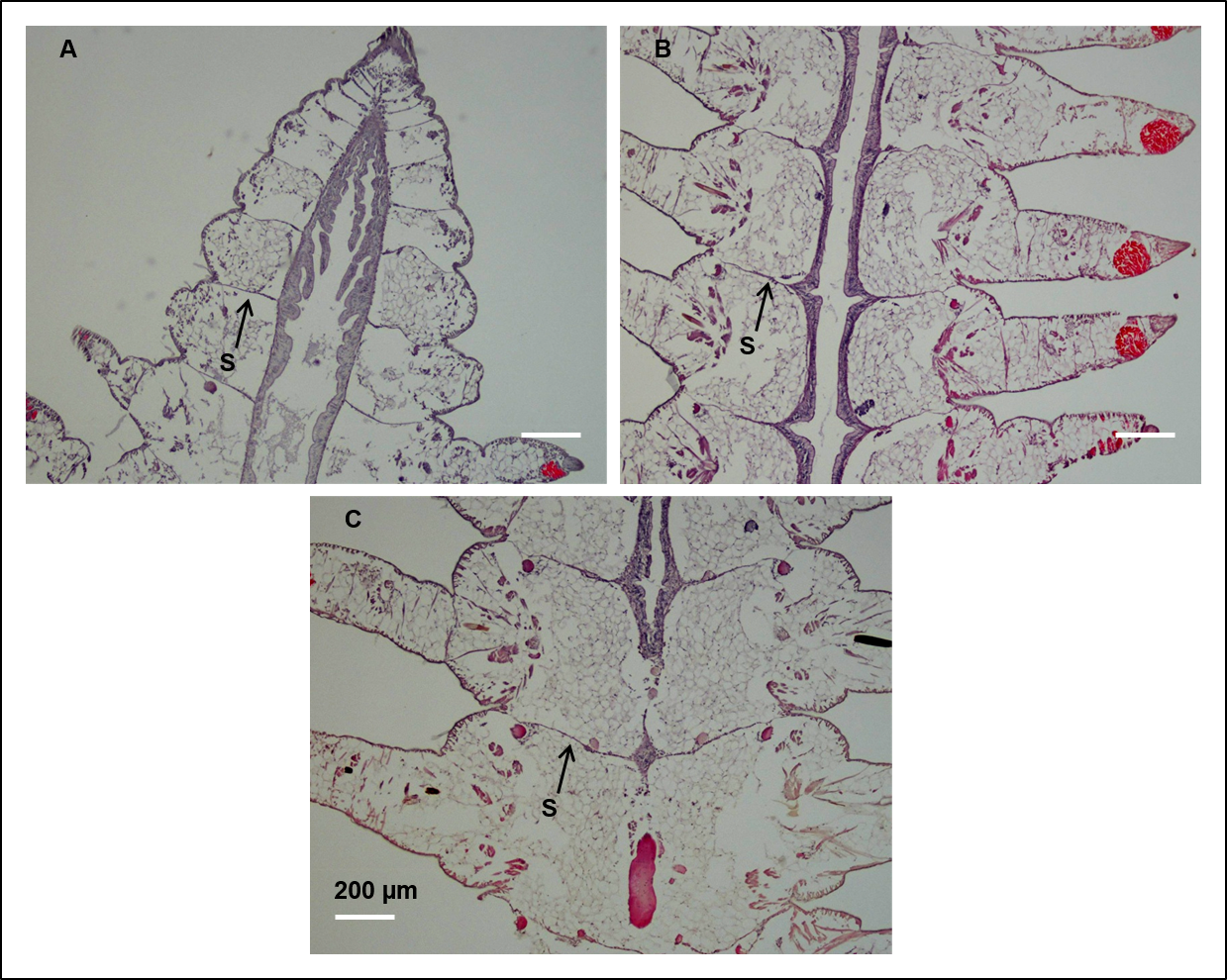

Supplement: Additional file 1: Figure S1. — The morphology of the Inter-segmental septa in the adult P. nuntia. Sections show the presence of complete inter-segmental septa (S) in the tail (A), mid-body (B) and anterior-body region (C). Coronal sections, 7 μm; hematoxylin and eosin stain. (PNG 2009 kb) [file 40851_2015_35_MOESM1_ESM.png]
